# Cognitive Development Trajectories in Preterm Children With Very Low Birth Weight Longitudinally Followed Until 11 Years of Age

**DOI:** 10.3389/fphys.2019.00307

**Published:** 2019-04-02

**Authors:** Sofia Ryytty Stålnacke, Mesfin Tessma, Birgitta Böhm, Eric Herlenius

**Affiliations:** ^1^Department of Women’s and Children’s Health, Karolinska Institutet, Karolinska University Hospital, Stockholm, Sweden; ^2^Department of Learning, Informatics, Management and Ethics – LIME, Karolinska Institutet, Stockholm, Sweden

**Keywords:** preterm (birth), development, cognitive stability, medical complications, academic achievements

## Abstract

**Background:** There is a high prevalence of cognitive dysfunction in very low birthweight (500–1250 g) infants (VLBW). Understanding long-term risk factors associated with cognitive development in preterm children requires longitudinal characterization. Thus, follow-up evaluations, including identification of risks and resilience influences–are important to promote health and cognitive abilities of children born preterm.

**Aim:** To examine changes in cognitive development from birth until 11 years of age in preterm children with very low birthweight.

**Methods:** 24 VLBW infants, at the Karolinska University Hospital, Stockholm, were assessed with regards to cognitive functioning at three times during development at 18 months, 5 and 11 years of age using standardized tests. Longitudinal data were analyzed using Generalized Estimating Equation (GEE) univariate and multivariate models.

**Results:** The follow-up rate was 100%. Level of cognitive functioning at 18 months and at 11 years was similar. Females had higher cognitive scores than males at all three timepoints. We found that intraventricular hemorrhage (IVH) and prolonged invasive ventilatory support (>7 days) had a negative effect on cognitive functioning. Higher levels of parental education had a favorable influence on cognitive functioning over time.

**Conclusion:** Level of cognitive development at 18 months was highly predictive of level of cognitive function at 11 years of age and differences in assessment scores between male and female VLBW infants persisted. Additional longitudinal studies, performed before school entry and across childhood, are needed to further elucidate the cognitive trajectories of preterm children.

## Introduction

Pre-term birth is associated with dysfunctional development of vital organs and increased risk of cognitive impairment later in life. Some problems appear during the first weeks of life and can be successfully treated, whilst others have a permanent influence on the development. Brain injury such as intraventricular hemorrhage (IVH) and periventricular leukomalacia (PVL) are associated with a high risk of neurodevelopmental disability (Volpe, 1980; Volpe et al., 2011). Preventing brain injury by supporting the respiratory control systems in the preterm infant is crucial. Apnea of prematurity can prolong the need for invasive ventilatory support and bronchopulmonary dysplasia, which are both associated with neurodevelopmental impairment (Janvier et al., 2004; Hofstetter et al., 2008; Doyle and Anderson, 2009). The degree of prematurity and the presence of comorbidities of more than one harmful factor influence the severity of developmental deficiencies in cognitive functioning, as well as in academic achievements (Schmidt et al., 2015). Inflammation has emerged as a critical contributor to both normal development and injury outcome in the immature brain (Hagberg et al., 2015). Neonatal factors found to predict a lower adulthood IQ include: respiratory distress syndrome, IVH, mechanical ventilation, mobility problems, parenteral nutrition, low to middle socioeconomic status of parents, and poor parent-infant relationship (Breeman et al., 2017). Furthermore, a range of perinatal vulnerability factors have been associated with male sex supporting the concept that male sex is an important biological risk factor in extremely preterm infants. Future prospects for preterm children are of utmost interest for parents, pediatric medicine, schools, and society (Moore et al., 2012; Aylward, 2014).

The results of cognitive assessments in children who were born very (week 28–32) or extremely (<week 28) preterm, range from severe and mild levels of intellectual disability to cognitive levels above average. The prevalence for severe cognitive delay is higher in populations of very (Murray et al., 2014) and extremely premature children (Johnson et al., 2009). Though the majority of preterm children perform within normal range of general cognitive functioning, as a group they perform 0.5–1 SD below that of full term children (Bohm et al., 2002; Rose et al., 2011; Luttikhuizen dos Santos et al., 2013; Linsell et al., 2018). Specific cognitive functions such as attention, working memory, and processing speed are also often delayed (Rose et al., 2012; Murray et al., 2014).

Early developmental assessment of cognition from 18 to 24 months post term age (corrected for prematurity) tend to be stable in preterm children with average cognitive development, but future cognitive functioning seems harder to predict when children are performing 1 to 2 SD below expectation, especially in VLBW infants (Roberts et al., 2010; Luttikhuizen dos Santos et al., 2013; Wong et al., 2016).

There is strong evidence that parental education acts as a predictor for cognitive development in preterm children (Bohm et al., 2002; Breeman et al., 2017). In addition, parental level of education, employment and income have additionally shown independent, and additive effects on cognitive gain across preschool years (Manley et al., 2015; Beauregard et al., 2018). Cognitive outcome after preterm birth is heterogeneous, and group level analyses may disguise individual variability in development.

Thus, long-term studies that address individual patterns and explore trajectories in cognitive development are emerging (Stalnacke et al., 2015; Wong et al., 2016; Mangin et al., 2017). A significant number of children born very preterm or VLBW experience difficulties in school. To be able to reduce the long-term risks associated with VLBW birth, an improved understanding of the mechanisms and risk factors placing these children at risk of cognitive delay and dysfunction is necessary. Identifying factors affecting the predictive accuracy of early neurodevelopmental assessments and individual trajectories of overall, as well as specific, cognitive function is important in order to enable earlier support and intervention.

### Aim

The aim of the present study was to investigate trajectories of cognitive functioning at the age of 18 months, 5 and 11 years in a Swedish cohort of preterm children with very low birth-weight (500–1250 g). The concordance over time in different aspects of cognition were studied as well as the differences within the cohort predicted by sex, preterm birth factors, medical risk factors, and parental level of education.

## Materials and Methods

The Swedish cohort is part of an international multisite follow-up study, the Caffeine for Apnea of Prematurity trial (CAP), a randomized and placebo-controlled study of the safety and efficacy of neonatal Caffeine citrate (Methylxanthines), for management and/or prevention of apnea in premature children with a birth-weight of 500–1250 g (Schmidt, 2005; Schmidt et al., 2011). Information about the random assignment is confidential to members of the double-blind CAP trial. Therefore, effects of drug treatment are not evaluated in this study.

### Participants

The present Swedish cohort consists of 24 VLBW infants, born at the Karolinska University Hospital between 2001–2004. They were enrolled in the study during the first week after birth and received caffeine therapy or placebo, until it was no longer needed during the neonatal period (Schmidt et al., 2006). The characteristics of the preterm infants and parental education is presented in Table 1.

**Table 1:** Characteristics of preterm infants and parents’ educational level, Swedish CAP cohort.

	Descriptive
Variables	statistics
**Infants Characteristics, *n* = 24**	
Birth weight, mean (SD), g	981 (167)
Gestational age, mean (SD), week	27 (1.1)
Female, no. (%)	10 (42)
Very preterm (28–29 weeks), no. (%)	6 (25)
Extremely preterm (<28 weeks), no. (%)	18 (75)
SGA, no. (%)	8 (33)
Singleton birth, no. (%)	20 (83)
Respiratory support <8 days no. (%)	7 (25)
Invasive ventilatory support <6 days no. (%)	13 (54)
Invasive ventilatory support >7 days no. (%)	4 (17)
**Medical complications**	
CLD, no. (%)	4 (17)
BPD, no. (%)	7 (29)
IVH, no. (%)	5 (21)
ROP, grade >3, treated, no. (%)	4 (17)
ROP, grade 1–2, no. (%)	3 (13)
Sepsis, no. (%)	16 (67)
**Parental Education Level, at 11 years (*n* = 48)**	
Less than elementary school level yes, no. (%)	2 (4)
High school, yes, no. (%)	21 (45)
Diploma, yes, no. (%)	11(23)
Bachelor/masters, yes, no. (%)	11(23)
PhD, yes, no. (%)	2 (4)

*Respiratory support (Invasive and non-invasive ventilatory support combined) <8 days. CLD = chronic lung disease, BPD = bronchopulmonary dysplasia, IVH = intraventricular hemorrhage, ROP = retinopathy of prematurity*.

### Ethics Statement

The study was performed in accordance with European Community guidelines. The regional ethics committees at the Karolinska Institutet and Stockholm County approved the study (2012/1401). Informed written consent was obtained from the parents. Feedback to parents was communicated after assessments.

### Procedure

Follow-up in terms of medical, motor, and cognitive assessment was performed three times. The cognitive assessment was performed by clinical psychologists at 18–24 months and at the 5th and 11th years of age. The assessment at 18 months, at 5 years as well as at 11 years were corrected for preterm birth. Child and parent ratings of behavior was collected at 11 years: Presentation of motor assessments and behavior ratings have been planned for in the near future.

### Tests and Measures

General cognitive development/functioning was estimated at the three assessments points, with the second edition of the Bayley Scales of Infant Development, (BSID-II), mental development index (MDI), WPPSI-III full scale index (FSIQ), and WISC-IV full scale index (FSIQ), respectively. A validated WISC-IV-short form was used (Crawford et al., 2010) in exchange for WASI-II and DLS Swedish Reading and Spelling tests replaced the corresponding sub-tests from WRAT-4. Standard scores on cognitive indexes have a mean of 100 and standard deviation (SD) of 15, with higher scores indicating a higher level of cognitive development/functioning (Bayley, 1993; David, 2006, 2007). Tests and measures evaluated in the study are presented in Table 2.

**Table 2:** Cognitive test during follow up.

Age	Test methods	Measures
18 month	BSID-II, Bayley scales of infant development – second edition	Mental development index (MDI)
5 year	WPPSI-III, Wechsler’s Preschool and Primary Scale of Intelligence - Third edition	Full scale IQ (FSIQ): verbal function (VI) visual function (PI) and processing speed (SI)
5 and 11 year	Beery -Buktenica Developmental Test of Visual-Motor Integration (VMI) – sixth edition	Visuomotor integration (VMI), visual perception (VP) and fine motor coordination (FMC)
11 year	WISC-IV, Wechsler’s Intelligence Scale for Children – fourth edition, short form	Full scale IQ (FSIQ): 7 subtests. Verbal function index (VI): subtests similarities and vocabulary. Performance index (PI): subtests block design and matrix reasoning. Index speed of process (SI): subtests coding and symbol search. Working memory: subtest digit span
	RCFT: rey complex figure test and recognition trial	Copy score and Recall score
	TEA-Ch; test of everyday attention for children	Selective (subtest sky search), sustained (subtest score), divided (subtest sky search dt) and shifting (subtest creature)
	WRAT-4: the wide range achievement test	Mathematics (numerical operations)
	DLS for school year 4–6; diagnostic material for reading and writing. Swedish tests	Spelling 36 words and Reading 47 words

### Statistical Methods

Descriptive statistics are presented either as means, SD, and medians (ranges) for continuous data, or as frequencies or percentages for categorical variables.

For continuous variables the paired *t* test was used to examine within subject changes between two assessment time points and the independent *t* test was applied to examine difference between males and females. The Mann-Whitney test was employed when there was a violation of the assumptions of normality and equal variance.

Based on the three indexes, BSID-II (MDI), WPPSI-III (FSIQ), and WISC-IV (FSIQ) a standardized cognitive scale was created (-2 to + 2). The Friedman ANOVA was performed to compare the three scales considering the ordinal property of the outcome variable. *Post hoc* analysis with Wilcoxon signed-rank tests was conducted with a Bonferroni correction applied since the overall Friedman ANOVA was significant.

We employed Generalized Estimating Equation (GEE) to analyze the change over time and to examine the fixed effects of male/female sex. A time x sex interaction term was introduced in the model to examine heterogeneity effect. GEE was also performed to control for potential confounders. Explanatory variables were selected based on clinical relevance, earlier research findings and univariate GEE models. Upon completion of the univariate analyses, we selected variables for the multivariate analyses including factors judged to be potential confounders. We have also employed Linear mixed model (LMM) to examine the relationship between the response IQ score as numerical continuous outcome variable and clinical and demographic explanatory variables. Both GEE and LMM simultaneously examine the relationship between each predictor and the outcome variable and the relationship between changes in the predictors and changes in the dependent variable. The dependent variable cognitive level was coded as -2, -1, 0, 1, and 2 for each timepoint, based on the standardized cognitive level which represent severe delay (≤ = 70), moderate delay (71–85), normal (86–114), high (115–130), and superior (≥131) cognitive levels, respectively. Categorical explanatory variables were coded depending on their level. If only two levels existed, the reference category was the category with the higher code number: The variable sex was coded as female = 1 and male = 2, thus male was the reference category. The categorical variable “educational level” constituted six levels, which were coded as 1 = less than elementary, 2 = elementary, 3 = high school, 4 = diploma, 5 = university degree (Bachelor or Masters), and 6 = Ph.D. holder. Less than elementary was the reference category. IVH was coded as 0 = no IVH, 1 = Grade 1, 2 = Grade 2; grade 2 was the reference category. Time was coded as 1 = 18 months, 2 = 5 years, and 3 = 11 years (the reference category). Level of respiratory support was coded as: 0 = Invasive (endotracheal tube *in situ*) and non-invasive (continuous positive airway pressure- CPAP) ventilatory support combined <8 days, 1 = mild (combined ventilatory support >8 days but invasive ventilatory support ≤6 days) and 2 = prolonged (invasive ventilatory support >6 days). The results of GEE are presented as estimate (regression coefficient), standard error (SE), Wald Chi-square value and *p*-value. Results of LMM are included in Supplementary Table 1. SPSS version 25.0 (IBM, NY, United States) was used for all data analyses and Statistica 13 for case profile graphical presentation. The level of significance was specified as *p* = 0.05.

## Results

**Figure 1:**
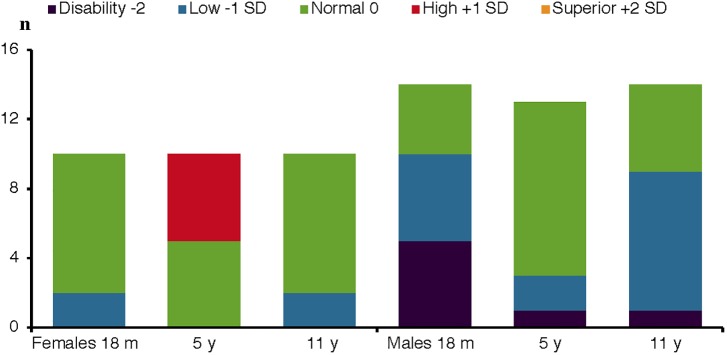
Cognitive level for females and males at three assessment points. Cognitive index scores standardized in levels. Index score <70 = disability/severe delay. 71–85 = low/moderate delay. 86–115 = normal/average. 116–130 = high. <131 = superior. Assessment at 18 months, 5 and 11 years of age, 10 female and 14 male preterm VLBW children.

**Figure 2:**
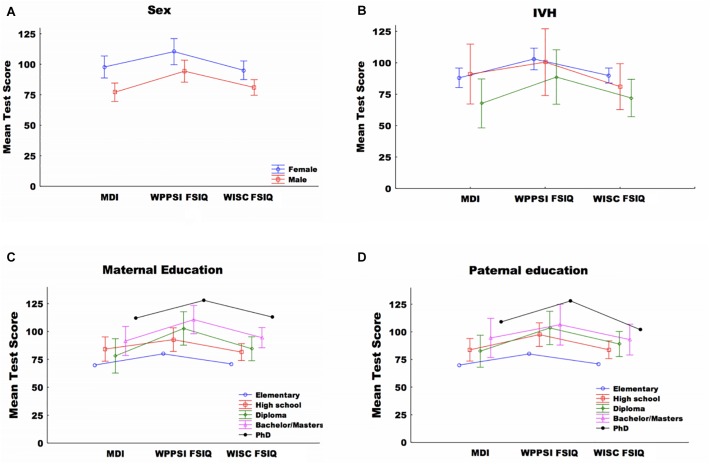
**(A)** Mean test score by sex and test type. **(B)** Mean test score by IVH and test type. **(C)** Mean test score by maternal educational level and test type, **(D)** mean test score by paternal educational level and test type. Mental development index (MDI), WPPSI-III Full scale index (FSIQ), and WISC-IV Full scale index (FSIQ) 18 month, 5 and 11 years, respectively. Data is presented as mean + SEM.

All 24 children in the Swedish cohort participated in all three assessments, thus the follow up was 100%. Table 1 shows the characteristics of preterm birth, medical and social background factors for the complete group as well as separated for by sex. At the 5-year and 11-year assessment, no child was deaf, blind or had cerebral palsy. Preterm medical risk variables that were considered to have too few participants to be included in further analyses are not shown in the tables. The level of the cognitive index score at the three assessment points for each gender are summarized in Figure 1. Table 2 shows all tests, including abbreviations, cognitive, and academic measures for the Swedish CAP cohort. The test results are summarized in Table 3. The mean test score of the study subjects is presented by sex, maternal education level, paternal education level, and IVH (Figure 2A–D, respectively). The independent sample *t*-test was employed to compare means for between group differences and the paired *t*-test for within-subject change (Table 4).

**Table 3:** Test results for the Swedish cohort.

	Swedish data:	Female	Male	Total *N* = 24
	*N* = 24	*N* = 10	*N* = 14	min.– max.
Tests	mean (SD)	mean (SD)	mean (SD)	Results
BSID-II MDI **18 months**	85.7 (17.0)	97.8 (15.1)	77.1 (12.7)	55 – 114
WPPSI-III FSIQ **5 year** (full scale)	102.0 (16.6)	110.4 (14.6)	95.9 (15.7)	57 – 128
WPPSI-III VI (verbal)	98.1 (17.8)	107.0 (15.9)	91.8 (16.8)	47 – 137
WPPSI-III PI (visual)	97.3 (12.9)	100.8 (11.8)	94.7 (13.4)	70 – 120
WPPSI-III SI (speed)	79.0 (12.8)	87.9 (12.6)	72.6 (8.7)	46 – 114
WISC-IV FSIQ **11 year**	86.9 (13.4)	95.1 (12.8)	81.0 (10.8)	56 – 113
WISC-IV VI (verbal)	89.0 (15.0)	98.9 (14.8)	86.2 (11.3)	70 – 122
WISC-IV PI (visual)	95.5 (12.0)	101.2 (12.9)	91.4 (9.8)	77 – 121
WISC-IV SI (speed)	89.4 (14.1)	95.6 (12.4)	84.9 (13.8)	55 – 116
Beery VMI **5 year** (copying**)**	96.3 (16.8)	102.9 (21.7)	91.6 (10.8)	70 – 132
VMI VP (perception)	105.9 (23.6)	111.1 (23.7)	102.1 (23.7)	45 – 132
VMI FMC (fine-motor control)	92.4 (21.0)	101.3 (21.7)	86.1 (18.8)	45 – 130
Beery VMI **11 year** (copying)	90.5 (15.1)	98.4 (10.4)	84.9 (15.7)	62 – 115
VMI VP (perception)	96.0 (17.3)	100.9 (16.1)	92.5 (17.7)	45 – 113
VMI FMC (fine-motor control)	87.8 (15.7)	95.1 (15.3)	82.6 (14.3)	45 – 114
WRAT-4, Mathematic	81.2 (16.4)	86.7 (16.4)	77.3 (9.7)	16 – 40
Reading 47 words; %	80.0 (21.0)	87.9 (14.1)	74.4 (23.7)	4 – 47
Spelling 36; stanine	3.3 (1.9)	4.4 (1.7)	2.5 (1.7)	3 – 32
TEA-Ch: selective att.	7.6 (3.3)	9.1 (3.3)	6.5 (2.9)	2 – 13
TEA-Ch: sustained	7.1 (2.1)	8.1 (2.2)	6.4 (1.8)	4 – 13
TEA-Ch: divided	5.3 (2.4)	5.4 (2.8)	5.2 (2.2)	1 – 10
TEA-Ch: shifting	9.3 (3.3)	10.2 (3.4)	8.6 (3)	3 – 13
WISC-IV digit span, forward	5.0 (1.0)	5.2 (1.3)	4.9 (0.8)	3 – 7
WISC-IV digit span, backward	3.6 (0.9)	3.8 (1.0)	3.4 (0.8)	2 – 6
RCFT, copy	24.3 (9.6)	28.8 (5.8)	21.1 (10.6)	3 – 36
RCFT, delayed recall	11.7 (7.9)	13.9 (5.0)	10.0 (9.4)	0 – 25.5

**Table 4:** Results of the paired and independent *t* tests of the Swedish cohort.

Scale	Mean difference (95% CI)	*P*-value
**Paired *t* test**		
2 VMI SS – VMI SS	-5.5 (-9.8, -1.3)	0.01
2 VMI VP – VMI VP	-10.1 (-17.0, -3.2)	<0.01
2 VMI Motor – VMI motor	-5.2 (-11.6, 1.1)	0.1
WISC VI – WPPSI VI	-13.1 (-17.3, -8.8)	<0.001
WISC PI – WPPSI PI	-8.8 (-12.6, -4.9)	<0.001
WISC SI – WPPSI S	10.4 (5.8, 15.6)	<0.001
**Independent *t* test (female – male)**		
2VMI SS	13.5 (1.6, 25.3)	0.03
2VMI VP	0.03 (-0.4, 0.5)	0.42
2VMI FMC	13.1 (0.5, 25.7)	0.04
WPPSI FSIQ	14.6 (1.6, 27.7)	0.03
WPPSI VI (verbal)	16.0 (1.1, 30.8)	0.04
WPPSI PI (visual)	6.5 (-4.9, 18.0)	0.25
WPPSI SI (speed)	17.2 (-4.9, 27.2)	<0.001
WISC FSIQ	14.1 (4.1, 24.1)	<0.01
WISC VI (verbal)	12.6 (1.7, 23.7)	0.03
WISC PI (visual)	9.8 (0.2, 19.3)	<0.05
WISC SI (speed)	10.7 (-0.7, 22.1)	0.17

*Mean differences, 95% confidence interval (CI) and *p*-values for paired *t* and independent *t* tests*.

### Cognitive Level Through Development

Friedman test revealed a statistically significant difference in the standardized cognitive index over time, *χ*^2^ = 18.7, df = 2, *p* < 0.001. *Post hoc* analysis with Wilcoxon signed-rank tests was conducted with a Bonferroni correction applied, resulting in a significance level set at *p* = 0.017. We observed a statistically significant difference between Bayley-II MDI (at 18 months) and WPPSI-III FSIQ (at 5 years) (*p* = 0.001) and between WPPSI-III FSIQ (at 5 years) and WISC-IV FSIQ (at 11 years) (*p* = 0.002). However, we did not observe significant differences between BSID-II MDI and WISC-IV FSIQ (*Z* = -1.67, *p* = 0.096). WISC-FI was significantly related to WISC-SI, Mathematics SS, sex, and IVH (Supplementary Table 1). LMM also revealed that time was a statistically significant predictor of cognitive score. We observed a statistically significant difference between WPPSI-III FSIQ (at 5 years) and WISC-IV FSIQ at 11 years (*p* = 0.02). However, we did not observe a significant difference between BSID-II MDI and WISC-IV FSIQ (*p* = 0.79).

The univariate and multivariable General Estimating Equation (GEE) analysis were carried out and are presented in Tables 5, 6 respectively. Sex was observed to be a predictor of standardized cognitive score. Female sex was positively associated with standardized score in all multivariable models, Table 6. Univariate GEE revealed that the likelihood of having a higher cognitive score was positively related to time at 5 years (*B* = 1.58, Wald Chi-square = 10.8, *p* = 0.001) with 11 years as a reference. However, when the dependent variable was the standardized Z score, GEE revealed that the likelihood of having a higher Z cognitive score was positively related to time (Wald Chi square 18.7, df = 2, *p* < 0.001). Higher Z score value was positively related to time at 5 years (*B* = 2.1, Wald Chi-square = 12.9, df = 1, *p* < 0.001) with 11 years as a reference.

**Table 5:** Results of GEE univariate analysis with parameter estimates, standard errors and Wald Chi square for the cohort for the dependent variable cognitive level.

			Wald
Variable	B	SE	Chi-square	*P*-value
Sex = Female	2.25	0.79	8.14	0.004
Time			16.2	<0.001
Time = 18 months	-0.41	0.31	1.78	0.18
Time = 5 years	1.58	0.48	10.8	0.001
SGA = 0	1.1	0.72	2.34	0.12
BPD = 0	-0.12	0.67	0.03	0.86
IVH			8.6	0.01
IVH = 0	2.4	0.93	6.45	0.01
IVH = 1	2.9	1.00	8.43	0.004
**Maternal education^∗^**		
University	5.5	0.86	40.6	<0.001
Bachelor/masters	3.4	0.83	16.5	<0.001
Diploma	1.7	0.42	16.3	<0.001
Elementary	1.9	0.72	7.2	0.007
**Paternal education^∗^**		
University	5.3	0.91	34.1	<0.001
Bachelor/masters	2.9	0.92	10.1	0.001
Diploma	3.8	0.59	41.6	<0.001
High School	2.2	0.42	27.4	<0.001
Elementary	1.9	0.67	7.8	0.005
**Respiratory^#^support level**				
Level = 0	1.7	0.70	5.5	0.019
Level = 1	1.6	0.65	5.8	0.015

^∗^*Reference: less than elementary. **^#^**Reference: respiratory support = level 2 (invasive ventilatory support >6 days)*.

**Table 6:** Results of GEE multivariable analysis with parameter estimates, standard errors and Wald Chi square for the cohort for the dependent variable cognitive level.

			Wald
Variable	B	SE	Chi-square	*P*-value
**Model 1 with SGA**		
SGA	0.82	0.76	1.2	0.28
Sex = female	2.51	0.91	7.51	0.006
Time = 18 months	-0.70	0.42	2.81	0.09
Time = 5 years	1.88	0.49	15.0	<0.001
**Model 2 with IVH**				
IVH = 0	1.6	1.08	2.12	0.14
IVH = 1	3.6	1.07	11.2	0.001
Sex = female	2.76	1.00	7.6	0.006
Time = 18 months	-0.75	0.47	2.5	0.11
Time = 5 years	2.01	0.52	14.7	<0.001
**Model 3^∗^ Maternal education**		
University	5.1	1.26	16.4	<0.001
Bachelor/masters	3.6	0.97	13.8	<0.001
Diploma	1.9	0.68	7.81	0.005
Elementary	1.5	0.90	2.8	0.094
Sex = female	2.68	0.96	7.77	0.005
Time = 18 months	-0.77	0.45	2.86	0.09
Time = 5 years	1.51	0.84	3.21	0.07
**Model 4^∗^ Paternal Education**		
University	4.51	1.22	12.1	<0.001
Bachelor/Masters	1.93	0.95	4.0	0.047
Diploma	2.43	1.25	3.78	
High school	2.60	0.72	13.4	<0.001
Elementary	1.74	0.78	4.91	0.027
Sex = female	2.53	1.26	3.93	0.047
Time = 18 months	-0.78	0.48	2.71	0.10
Time = 5 years	2.04	0.54	14.3	<0.001
**Model 5 Respiratory support^#^**		
Level = 0	3.15	1.25	6.3	0.012
Level = 1	2.35	1.25	3.5	0.06

^∗^*Reference: less than elementary; **^#^**Reference: respiratory support = level 2. The multivariable models include the variables sex and time with the interaction*.

General Estimating Equation showed that IVH was a predictor of standardized cognitive score. In addition, level of respiratory support was a significant predictor of standardized cognitive score in the univariate (*p* = 0.03) and multivariable GEE analysis when controlling for sex and time in the latter analysis (*p* = 0.04). SGA and BPD were not statistically significant in either univariate and multivariable GEE analyses, when controlled for sex and time, Tables 5, 6.

Univariate GEE analysis revealed a positive relationship between parental education levels and the standardized score (Table 5). Both maternal and paternal educational levels were positively associated with the standardized score in all multivariable models when controlling for sex and time (Supplementary Table 2). We did not observe interactions between time and other clinical and demographic factors in all used multivariable models.

The independent *t*-test revealed that there was statistically significant score differences in between the sexes for many items indicating higher score for girls compared to boys (Table 4). The paired mean differences were also statistically significant for most items (Table 4).

### Cognitive Functioning and Academic Achievement at 11 Years of Age

Data analyses revealed broad confidence intervals for results of specific cognitive measures (TEA-Ch, RCFT, and WISC-IV digit span) at 11 years of age. WISC-IV SI was found significantly lower than WISC-IV FSIQ at 11 years (*p* ≤ 0.001). Measures of academic achievement showed sex difference in Mathematic WRAT-4 (*p* = 0.04) and Spelling DLS measures (*p* = 0.01), favoring girls. We did not observe differences in reading ability.

## Discussion

This prospective cohort study has three assessment points of cognitive development and a follow-up rate of 100%, which adds stability to the results and bolster our conclusions. We found that, male sex and parental education had a significant impact on cognitive test results. This concurs with previous studies but the present data further underline the effect of sex and parental education in long term cognitive and academic outcomes (Bohm et al., 2002; Linsell et al., 2015, 2018; Mangin et al., 2017). IVH was identified as a strong predictor of cognitive outcome, and so was the cumulative duration of invasive ventilatory support. This is in accordance with recent studies, e.g., (Breeman et al., 2017). However, other medical complications (ROP, CLD/BPD, SGA, and sepsis) did not contribute to the explained variance. Sex differences were seen in all tests given, with females acquiring higher scores than males. Parental education was on average high in both mothers and fathers and all levels significantly influenced the cognitive outcome in the multivariate analyses.

The GEE is an appropriate statistical method to fit a marginal model for longitudinal data analysis since we have repeated measures over time. We measured 24 children at three time points with three different cognitive tests to examine their cognitive development/functioning. The repeated measurements thus provides a multivariate response of similar individuals. GEE is a common approach to longitudinal data based on population-averaged (marginal) approach. The GEE models the average response over the subpopulation sharing a common value of the predictors, as a function of the predictors (Liang and Zeger, 1986).

Cognitive z-scores from -2 to +2 are represented in this Swedish cohort of VLBW infants. GEE showed that the cognitive results were similar at 18 months and 11 years of age. We found the results to be important since predictability of early assessments varies. Robust findings based on meta-analyses and single studies imply that the predictability for later cognitive functioning in pre-school and school-aged children vary, from moderate in very preterm to poor in extremely preterm infants. In contrast, several studies of cognitive outcome between pre-school and middle-school age as well as adolescence, report stability in cognitive development (Stalnacke et al., 2015; Mangin et al., 2017).

Summarizing other studies of cognitive development from infancy to adolescence is challenging due to methodological deficiencies. Outcome studies often have only one or two assessment points, lack of a control group, loss to follow up, and a varied application of standardized test norms and statistical methodologies, rendering the conclusions of these studies hard to compare (Wong et al., 2016). Based on our study design and results we find it important to take into account that time points and time between cognitive assessments can affect findings in cognitive follow-up of preterm children. Of equal importance are longitudinal studies, and maintaining high retention throughout follow-up (Doyle and Anderson, 2018). Recently, the longitudinal EPI Cure study showed that cognitive test score in infancy and early childhood reflect early adult outcomes (Linsell et al., 2018).

The cognitive results at 5 years of age were markedly higher compared to cognitive levels at both 18 months and 11 years of age, which requires an account of potential methodological issues in the performance of said test. The Swedish WPPSI-III norms are based on British norms and validated in a limited sample of Swedish children (David, 2006). Thus, the high results at 5 years of age might be due to an incomplete Swedish validation of the British WPPSI-III norms. Test construction, validity and reliability issues of cognitive assessment in preterm children can have an impact on cognitive results and are also addressed in other studies (Luttikhuizen dos Santos et al., 2013; Spencer-Smith et al., 2015). The results are similar at 18 months and 11 years, both at individual and group level. Thus, the apparent increase at the 5-year assessment are likely due to test norm differences. Nevertheless, the heterogeneous nature of cognitive outcomes in individuals emphasize the importance of long-term follow-up and monitoring of infants born VLBW (Manley et al., 2015; Beauregard et al., 2018).

We know little about the developmental pace regarding different aspects of cognition, especially for preterm children, with their risk of suboptimal neurocognitive development/ cognition. There is still considerable debate and uncertainty with regards to whether very preterm children grow into or out of their cognition problems (Mangin et al., 2017). Our and other long-term studies, that address individual patterns and explore trajectories in cognitive development, indicate that cognitive test scores in infancy and early childhood may reflect cognition and academic performance during early school years (Stalnacke et al., 2015; Wong et al., 2016; Mangin et al., 2017; Linsell et al., 2018). Notably, cognitive trajectories may differ. Recently, several distinct language trajectories were revealed, in very preterm and full term infants examined at 2, 5, 7, and 13 years (Nguyen et al., 2018). This and our study underline the importance of monitoring cognition in children born very preterm before school entry and across childhood.

The process of cognitive maturation is complex, multidimensional and influenced by genetic predisposition, environmental factors and experience (Fuster, 2005) and ruptures in development (Anderson, 2001; Anderson et al., 2008). Effects on brain development (Haynes et al., 2011; Volpe et al., 2011) and coherent alternated cognitive trajectories (Mangin et al., 2017) are found in preterm children (Thompson et al., 2018). Comparing the cognitive results at 5 years and 11 years of age indicated both stability and change in the cohort. Level of verbal intelligence (VI) was significantly lower at 11 years and so was the visuo-constructive measure (Beery VMI). Perceptual intelligence (PI) was found to be stable. Processing speed (SI) was higher at 11 years. The visuo-constructive measure (Beery VMI) was significantly lower at 11 years. Declining results and differences between cognitive functions may be affected by specific deficits especially in executive functions. In our study it was not possible to draw a conclusion from executive tests at 11 years since the single specific cognitive measures, TEA-Ch, RCFT, and Digit Span, had broad confidence intervals and in combination with the effect of the cohort size could lead to random results. Problems with attention, working memory and processing speed are significantly more present among preterm children and tend to emerge during development (Rose et al., 2011, 2012; Murray et al., 2014). Deficits in attention and processing speed are identified as important abilities contributing to lower level of cognitive intelligence in preterm children (Rose et al., 2009, 2011, 2012). Cognitive functioning in preterm children is of great importance for school performance. Results in academic achievement are of special interest, as they will show any issues that may be a problem in school. Our test battery included mathematics, reading and spelling. Mathematics were significantly correlated to FSIQ at 11 years (*r* = 0.77) and WISC PI, WISC SI, and IVH-2 were found to significantly explain most of the variance (*R*^2^ = 0.64) of the dependent variable Mathematics.

Sex differences were seen in all tests given, with females acquiring higher scores than males. Except for the VMI visual perception (VP) and fine motor coordination, this was also consistent between the two assessment points. Females also performed higher in academics, mathematics and spelling. Male sex is known to be a disadvantage with regards to mortality, morbidity and incidence of brain injury in preterm children (Marlow et al., 2005; Hintz et al., 2006; Skiold et al., 2014). The major type of brain injury involves cerebral white matter and the principal cellular target is the developing oligodendrocytes (Volpe et al., 2011). Neonatal white matter abnormalities are associated with cognitive impairment across childhood (Mangin et al., 2017). The view of male sex as a risk factor for cognitive development varies. Extremely premature boys have an increased risk of lower cognitive outcome (Linsell et al., 2018) and of developing severe cognitive disability (Marlow et al., 2005). However, in very preterm children the difference based on male sex decreases with age and environmental factors become more significant (Linsell et al., 2015; Mangin et al., 2017). The present results are in line with previous as well as recent studies (Doyle et al., 2015; Linsell et al., 2018; Thompson et al., 2018) and underlines the importance of considering sex in the potential cognitive developmental of preterm infants. Structural asymmetries and sexual brain dimorphism already exist at 1 month after birth in healthy term infants (Dean et al., 2018). In general, males have larger total brain volume and volumes differ by sex in regionally specific brain regions. We speculate that the different rates of maturation between the sexes renders the male brain, and hence male sex, a risk factor for long term cognitive outcome especially in VLBW preterm infants. Some of the underlying mechanisms for sex differences found, are delayed myelinization in preterm males, lower white matter volumes in males and differences in cerebral white matter microstructure compared to preterm females (Constable et al., 2008; Skiold et al., 2014).

We cannot exclude the effect of neonatal caffeine therapy from our findings. However, no adverse long-term effects of caffeine on development have been shown in the CAP studies, e.g., (Schmidt et al., 2006, 2017; Mürner-Lavanchy et al., 2018). The CAP-trial indicated a positive developmental trend in cognitive scores, between 18 months and 5 years of age, independent of treatment. Thus, our data are consistent with the results from this larger cohort (Schmidt et al., 2012). However, in the present cohort, the data and trajectories suggest that the increase was temporary and likely due to test norm differences. Nevertheless, cognitive results in our cohort indicate similar levels between 18 months and 11 years of age. Thus, on a group level, this does not support the suggestion that cognitive outcomes for preterm VLBW infants may improve throughout childhood (Ment et al., 2003). However, they are in line with, and underscore previous findings with regards to the importance of IVH, level of ventilatory support, sex as well as parental education for the long-term cognitive and academic outcomes (Doyle et al., 2015). These data emphasize the importance of early and repeated individual assessment enabling early intervention as well as adequate support during early childhood into adolescence, especially for those with cognitive delay and at risk for cognitive decline (Stalnacke et al., 2015; Mangin et al., 2017).

A strength of the present study is the 100% follow-up rate. The small group size is a limitation, which is why all medical variables and possible background variables could not be included in the model. The specific cognitive measures at 11 years of age, were found to have broad confidence intervals and thus in combination with the effect of cohort size these results could be random.

In summary analyzing measures of cognitive development across time better clarify changes and risk factors. We suggest long-term study designs, with more than two assessment points and with a substantial time between follow-up. It is possible to explore trajectories of cognitive function in a small cohort, when the cohort remains the same. Exploring individual patterns of cognition and brain development, as well as the underlying mechanisms associated with these, is necessary to increase knowledge about the maturation of preterm children. Cognitive development extends well beyond adolescence; and thus future follow-up should continue beyond 11 years. Early identification of children in need of support to promote development is an imperative necessity, especially for those with cognitive delay and at risk for cognitive decline.

## Data Availability

All datasets generated for this study are included in the manuscript and/or the Supplementary Files.

## Author Contributions

EH and BB conceptualized and designed the study. EH, SS, and BB acquired the data and revised the manuscript. All authors analyzed the data, drafted a significant portion of the manuscript or figures, and accepted the final version of the manuscript.

## Conflict of Interest Statement

The authors declare that the research was conducted in the absence of any commercial or financial relationships that could be construed as a potential conflict of interest.
