# Supplementary material for: Cognitive Development Trajectories in Preterm Children With Very Low Birth Weight Longitudinally Followed Until 11 Years of Age
Source: Front Physiol. 2019 Apr 2;10:307. doi: 10.3389/fphys.2019.00307 (PMC6454032; doi:10.3389/fphys.2019.00307)
Supplement: Supplementary file 1 [file Table_1.docx]

**Supplementary Table 1.** **Results of multiple linear regression** with R square, parameter estimates and 95% confidence interval for the dependent variable WISC full scale IQ (FSIQ).

| **Model Summary** | | | | |
| --- | --- | --- | --- | --- |
| **Model** | **R** | **R Square** | **Adjusted R Square** | **Std. Error of the Estimate** |
| 1 | 0,957^a^ | 0,915 | 0,898 | 4,296 |
| **a. Predictors: (Constant), IVH, Math SS Ord, Sex, WISC Speed I Ord** | | | | |

| **Coefficients^a^** | | | | | | | | |
| --- | --- | --- | --- | --- | --- | --- | --- | --- |
| Model | | **Unstandardized Coefficients** | | **Standardized Coefficients** | **t** | **Sig.** | 95,0% Confidence Interval for B | |
|  |  | **B** | **Std. Error** | **Beta** |  |  | Lower Bound | Upper Bound |
| 1 | (Constant) | 109,68 | 3,07 |  | 35,73 | 0,000 | 103,25 | 116,10 |
|  | WISC Speed I Ordinal | 7,34 | 1,57 | 0,36 | 4,67 | 0,000 | 4,05 | 10,63 |
|  | Maths SS Ordinal | 10,10 | 1,35 | 0,62 | 7,48 | 0,000 | 7,28 | 12,93 |
|  | Sex | -5,43 | 1,98 | -,20 | -2,74 | 0,013 | -9,58 | -1,29 |
|  | IVH | -2,89 | 1,29 | -,15 | -2,23 | 0,037 | -5,60 | -,188 |
| **a. Dependent Variable: WISC FSIQ** | | | | | | | | |
