# Supplementary material for: Cognitive Development Trajectories in Preterm Children With Very Low Birth Weight Longitudinally Followed Until 11 Years of Age
Source: Front Physiol. 2019 Apr 2;10:307. doi: 10.3389/fphys.2019.00307 (PMC6454032; doi:10.3389/fphys.2019.00307)
Supplement: Supplementary file 2 [file Table_2.docx]

Supplementary Table 2. Results of linear mixed model analysis with parameter estimates, standard errors and t-values for the cohort.

| **Variable** | **B** | **SE** | **t-value** | **P-value** |
| --- | --- | --- | --- | --- |
| **Model 1 with SGA** | | | | |
| SGA | 0.26 | 0.15 | 1.71 | 0.09 |
| Sex= Female | 0.62 | 0.17 | 4.25 | <.001 |
| Time =18 months | -0.29 | 0.17 | -1.21 | 0.23 |
| Time= 5 years | 0.42 | 0.15 | 2.43 | 0.18 |
| **Model 2 with IVH** |  |  |  |  |
| IVH = 0 | 0.56 | 0.21 | 2.59 | 0.01 |
| IVH= 1 | 1.06 | 0.29 | 3.59 | 0.001 |
| Sex= Female | 0.63 | 0.15 | 4.28 | <.001 |
| Time =18 months | -0.21 | 0.16 | -1.29 | 0.20 |
| Time= 5 years | 0.41 | 0.16 | 2.59 | 0.01 |
| **Model 3 *** | | | | |
| Maternal Education | | | | |
| University | 1.06 | 0.49 | 2.20 | 0.03 |
| Bachelor/Masters | 0.93 | 0.36 | 2.60 | 0.01 |
| Elementary | 0.61 | 0.36 | 1.70 | 0.09 |
| < Elementary | 0.50 | 0.35 | 1.40 | 0.16 |
| Sex= Female | 0.60 | 0.14 | 4.10 | <0.001 |
| Time =18 months | -0.21 | 0.16 | -0.21 | 0.21 |
| Time= 5 years | 0.42 | 0.16 | 2.54 | 0.01 |
| **Model 4 *** | |  |  |  |
| Paternal Education | |  |  |  |
| University | 1.02 | 0.51 | 2.00 | 0.05 |
| Bachelor/Masters | 0.60 | 0.40 | 1.50 | 0.14 |
| Diploma | 0.69 | 0.51 | 1.35 | 0.18 |
| High school | 0.84 | 0.37 | 2.26 | 0.03 |
| Elementary | 0.55 | 0.36 | 1.53 | 0.13 |
| Sex= Female | 0.64 | 0.17 | -3.91 | <.001 |
| Time =18 months | -0.21 | 0.17 | 1.22 | 0.23 |
| Time= 5 years | 0.42 | 0.17 | 2.44 | 0.02 |

* Reference: Less than elementary
